# Chromium(III) and iron(III) inhibits replication of DNA and RNA viruses

**DOI:** 10.1007/s10534-017-0027-9

**Published:** 2017-06-13

**Authors:** Sylwia Terpiłowska, Andrzej Krzysztof Siwicki

**Affiliations:** 1 Laboratory of Environmental Biology, Institute of Environmental Engineering, The John Paul II Catholic University of Lublin, Racławickie 14 Av, 20-950 Lublin, Poland; 2 Department of Microbiology and Clinical Immunology, University of Warmia and Mazury in Olsztyn, Oczapowskiego 13, Str., 10-957 Olsztyn, Poland

**Keywords:** Chromium(III), Iron(III), BVDV, HSV-1

## Abstract

The aim of this study was to examine the effect of treating of chromium(III) and iron(III) and their combinations on Herpes Simplex Virus type 1 (HSV-1) and Bovine Viral Diarrhoea virus (BVDV) replication. The antiviral efficacies of chromium(III) and iron(III) on HSV-1 and BVDV were evaluated using Real Time PCR method. Moreover, the cytotoxicity of these microelements was examined using the MTT reduction assay. The IC_50_ (50% inhibiotory concentration) for the chromium chloride was 1100 μM for Hep-2 cells and 1400 μM for BT cells. The IC_50_ for the iron chloride was 1200 μM for Hep-2 cells and more than1400 μM for BT cells. The concentration-dependent antiviral activity of chromium chloride and iron chloride against HSV-1 and BVDV viruses was observed. In cultures simultaneously treated with (1) 200 μM of CrCl_3_ and 1000 μM of FeCl_3_, (2) 1000 μM of CrCl_3_ and 200 μM of FeCl_3_, (3) 400 μM of CrCl_3_ and 800 μM of FeCl_3_, (4) 800 μM of CrCl_3_ and 400 μM of FeCl_3_ a decrease in number of DNA or RNA copies was observed compared with control cells and cells incubated with chromium(III) and iron(III) used separately. The synergistic antiviral effects were observed for chromium(III) and iron(III) against HSV-1 and BVDV.

## Introduction

The herpesviruses comprise a very large group of animal viruses, each one of which is usually specialized in nature to infect a particular species of mammal, marsupial, fish, bird, reptile, amphibian or even bivalve. All the studied herpesviruses share the three following characteristics: (1) typical morphology, (2) possession of a large genome consisting of single molecule of double stranded DNA ranging in size between 120 and 259 kbp, (3) the ability to follow productive infection to produce disease, as well as enter a latent phase in some cells of the infected natural host. This latent phase ensures survival of the viral genome throughout the lifetime of the particular infected individual and the ability to reenter the productive phase from time to time (Subak-Sharpe and Darga 1998).

The Herpes Simplex Virus type 1 (HSV-1) is a widely prevalent DNA virus that causes a range of human diseases. The most potent and well-studied anti HSV-1 agent is Acyclovir (ACV). However, ACV’s anti-HSV ability is attenuated when initial treatment is delayed. Furthermore, in immunocompromised individuals, ACV-resistant HSV-1 strains often arise (He and Tam 2010).

The Bovine Viral Diarrhoea Virus (BVDV), a member of the genus *Pestivirus* in the *Flaviviridae* family, is associated with various diseases of cattle, including respiratory infections, gastrointestinal infections, and reproductive problems such as infertility, abortion, still birth, and weak calves. The BVDV has been reported in other animals, e.g. pigs, sheep, goats, deer, and in captive and free-living ruminants. The BVDVs are grouped into two genotypes: BVDV1 and BVDV2. The BVDV2 can be subdivided into two subgenotypes, whereas the BVDV in North America can be subdivided into two genotypes: BVDV1a and BVDV1b, though more than 11 subgenotypes were reported in Europe (Kim et al. 2009).

Symptoms of acute infection range from an inapparent to a severe course and can involve the respiratory, enteric, reproductive, immune and endocrine systems (Letellier and Kerkhofs 2003; Zhang et al. 2014). The infection can cause a decrease in milk production, a reduced reproductive performance and growth retardation (Chai et al. 2013). Infection in the early gestation period with BVDV may produce persistently infected calves that are responsible for the spread of virus in the herds (Kim et al. 2009).

The BVDV, has been a good model virus for investigating HCV, which is a member of genus *Hepacivirus*, which belongs to the same family (Chai et al. 2013).

Therefore, the development of new anti-HSV and BVDV agents is important (He and Tam 2010). Currently, there is no specific antiviral agent directed against BVDV (Chai et al. 2013).

Nutrition plays an important role in the development and also in the prevention of cancer, cardiovascular diseases, and diabetes. Chromium(III) and iron(III) are trace elements necessary for growth and normal functioning of cells. Moreover, it has been found that zinc inhibits rhinovirus 3C protease, consequently inhibiting viral replication in vitro (Haase et al. 2008). Moreover, another study has shown, that inorganic selenium species inhibits in vitro replication of Coxackie virus B5 strain (Cermelli et al. 2002). The aim of this study was to examine the effect of chromium(III) and iron(III) used separately and simultaneous treating of chromium(III) and iron(III) on HSV-1 an BVDV replication. The concentrations of chromium chloride and iron chloride for these studies were chosen on the basis of other reports (Mazzotti et al. 2001, 2002) and our earlier investigations (Terpiłowska and Siwicki 2009, 2010, 2012).

## Materials and methods

### Chemicals

The Eagle’s Minimum Essential Medium (EMEM), the Dulbecco’s Modified Eagle’s Medium (DMEM), the heat-inactivated Fetal Bovine Serum (FBS) and the Horse Serum (HS) were purchased from The American Type Culture Collection (USA); Antibiotic/antimycotic solution (10,000 U/ml of penicillin, 10 mg/ml of streptomycin, 25 μg/ml of amphotericin B) and The MTT reduction assay—In vitro Toxicology Assay Kit MTT based (TOX-1) were purchased from Sigma Aldrich (Sigma Aldrich Inc., St. Louis, MO, USA); iron chloride (FeCl_3_ × 6H_2_0) and chromium chloride (CrCl_3_ × 6H_2_0) were purchased from Acros Organics (Belgium). Pathogenic Free DNA isolation Kit, Free RNA isolation Kit and Herpes Simplex Virus (HSV1/2) PCR Kit were purchased form Gene Proof (Czech Republic). Quantification of Bovine Viral Diarrhoea Virus Kit was purchased form PrimerDesign, Ltd. (United Kingdom).

### Cells and virus strains

Human epithelial carcinoma HEp-2 cells (ATTC CCL-23) and Herpes Simplex Virus type1 (ATTC VR-1493) were purchased from the American Type Culture Collection (USA). Hep-2 cells were cultured in EMEM supplemented with 10% (v/v) FBS, Antibiotic/antimycotic solution (1 ml per 100 ml of cell culture medium) at 37 °C in humidified atmosphere of 5% CO_2_/95% air.

BT (turbinate) cells (ATTC CRL-1390) and Bovine Viral Diarrhea Virus (ATCC VR-534) were purchased from the American Type Culture Collection (USA). BT cells were cultured in DMEM supplemented with 10% (v/v) HS, Antibiotic/antimycotic solution (1 ml per 100 ml of cell culture medium) at 37 °C in humidified atmosphere of 5% CO_2_/95% air.

### Cytotoxicity assay

Analyses of the in vitro cytotoxicity of chromium(III) and iron(III) were performed with an enzymatic assay (MTT assay) which is capable of quantifying the activity of mitochondrial enzymes in active and dividing cells. The MTT reduction assay is based on the enzymatic conversion of MTT (3-[4,5-dimethylthiazol-2-yl]-2,5-diphenyltetrazolium bromide) by the metabolically active cells in mitochondria. As a result, the MTT assay is a marker of mitochondrial function. The Hep-2 or BT cells were cultured at 2 × 10^5^ cells/ml as adherent monolayers in plastic tissue-culture dishes in EMEM or DMEM, respectively, supplemented with 10% (v/v) heat-inactivated FBS or HS, respectively, and Antibiotic/antimycotic solution (1 ml per 100 ml of cell culture medium). Cells were maintained at 37 °C in humidified incubator in atmosphere containing 5% CO_2_. Cells were used for cytotoxicological assay during exponential phase of growth. After 24 h of incubation the medium was exchanged for the fresh medium supplemented with chromium chloride or iron chloride at concentrations of 100, 200, 400, 600, 800, 1000, 1200 and 1400 μM. All concentrations mentioned above are the final concentrations in the incubations. After 24 h of incubation the MTT reduction assay was performed according to the original manufacturer’s instruction by Sigma-Aldrich—In vitro Toxicology Assay Kit MTT based (TOX-1). Results for all experiments represent triplicate determinations.

### Detection of anti-HSV-1 activity

The HEp-2 cells were cultured as adherent monolayers in EMEM supplemented with 10% (v/v) FBS and Antibiotic/antimycotic solution (1 ml per 100 ml of cell culture medium). After 24 h incubation the medium was exchanged respectively for fresh medium (control) or medium supplemented with chromium chloride or iron chloride at concentrations of 200, 400, 600, 800 and 1000 μM and for simultaneous treatment with chromium chloride at concentrations of 200, 1000, 400 and 800 μM and iron chloride at concentrations of 1000, 200, 800, 400 μM respectively. Two hundred microliters of virus suspension was added at rate of 10 TCID_50_/0.2 ml on Hep-2 to all treatments. After 48-hour of incubation DNA isolation was performed according to the original manufacturer’s instruction by the PathogenFree DNA Isolation Kit (GeneProof) and stored at −20 °C until needed. Viral nucleic acid was isolated from 200 μl tissue culture supernatant. The thus obtained DNA was analyzed by Real Time PCR Herpes Simplex Virus (HSV1/2) PCR Kit for 45 cycles with Rotor Gene Q (Corbett Life Science)-Amplification program: UDG decontamination 37 °C (2 min.), initial denaturation 95 °C (10 min.), denaturation 95 °C (5 s), annealing 60 °C (40 s)—reading for fluorescence signal, extension 72 °C (20 s). Results for all experiments represent triplicate determinations.

Real Time fluorescent measurements were obtained and the threshold cycle (C_t_) value for each sample was calculated by determining the point at which fluorescence exceeded a threshold limit. A standard graph of the C_t_ values obtained in series diluted external DNA standard was prepared. C_t_ values obtained from the samples were plotted on the standard curve, and the number of copies was calculated automatically by the Rotor-Gene Q Series Software 2.0.2 (Build 4).

### Detection of anti-BVDV activity

The BT cells were cultured as adherent monolayers in DMEM supplemented with 10% (v/v) HS and Antibiotic/antimycotic solution (1 ml per 100 ml of cell culture medium). After a 24 h incubation the medium was exchanged respectively for fresh medium (control), or medium supplemented with chromium chloride or iron chloride at concentrations of 200, 400, 600, 800 and 1000 μM and for simultaneous treatment with chromium(III) at concentrations of 200, 1000, 400 and 800 and iron(III) at concentrations of 1000, 200, 800, 400 respectively. Two hundred microliters of virus suspension was added at the rate of 10 TCID_50_/0.2 ml on BT cells to all treatments. After 5 days of incubation RNA isolation was performed according to the original manufacture’s instruction and stored at −20 °C until needed. Viral nucleic acid was isolated from 200 μl tissue culture supernatant. Isolation was performed according to the original manufacturer’s instruction by the PathogenFree RNA Isolation Kit (GeneProof). The thus obtained RNA was analyzed by Real Time PCR. The test was performed according to the original manufacturer’s instruction by the Quantification of Bovine Viral Diarrhoea Virus Kit (Primer Desing).

One step qRT-PCR combines the reverse transcription and real-time PCR reaction in a simple closed tube protocol. Amplification program: reverse transcription—10 min, 55 °C, enzyme activation—2 min, 95 °C, denaturation—10 s, 95 °C-50 cycles, data collection: 60 secs, 60 °C—50 cycles. Fluorogenic data was collected through the FAM and VIC channels. Results for all experiments represent triplicate determinations.

RT fluorescent measurements were obtained and the threshold cycle (C_t_) value for each sample was calculated by determining the point at which fluorescence exceeded a threshold. A standard graph of C_t_ values obtained was plotted on the standard curve, and the number of copies was calculated automatically by the Rotor-Gene Q Series Software 2.0.2 (Build 4).

### Statistical analysis of the data

The results were expressed as mean ± SD and the data were analyzed by the use of one way analysis of variance (ANOVA) with Tukey’s multi-comparison post-test using Statistica programme. In all the cases, p < 0.05 was considered significant.

## Results

### Cytotoxicity of chromium chloride and iron chloride on HEp-2 and BT cells

**Fig. 1:**
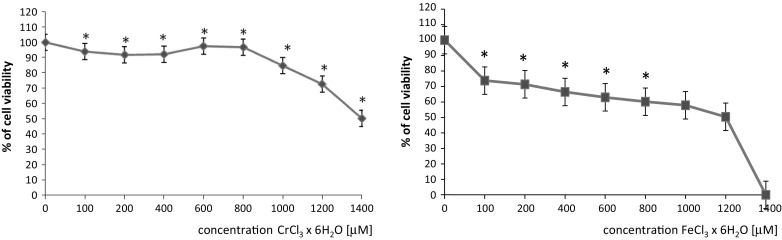
Cytotoxic effect of chromium chloride or iron chloride in HEp-2 cell line, detected with the MTT reduction assay. Values are given as percentage of cell viability. *p < 0.05, significance of difference compared with control

**Fig. 2:**
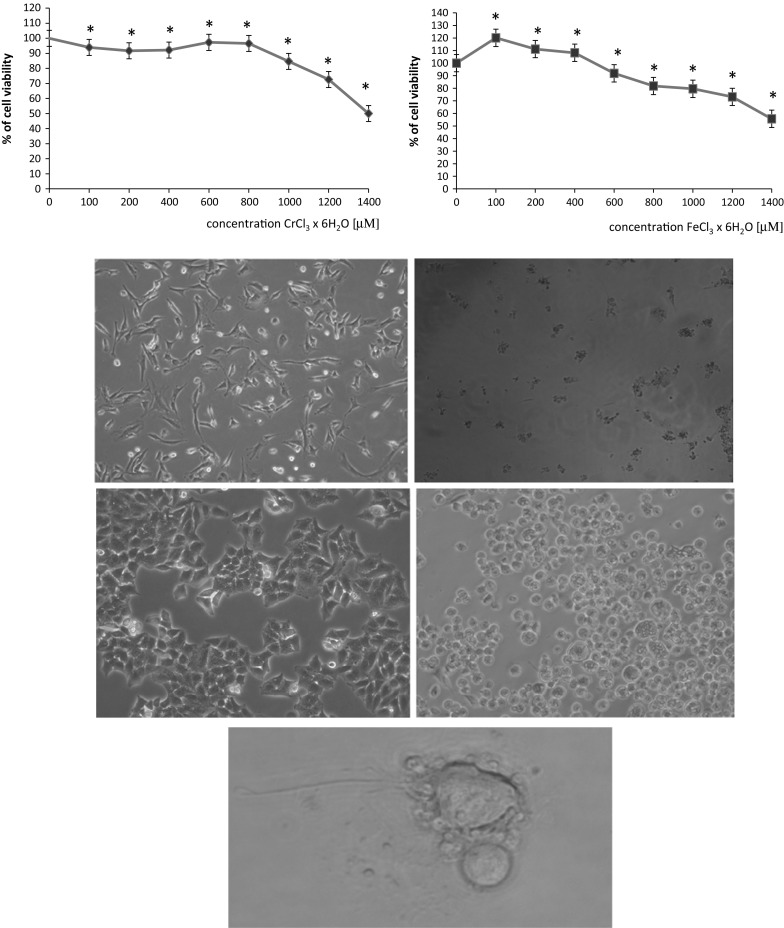
Cytotoxic effect of chromium chloride or iron chloride in BT cell line detected with the MTT reduction assay. Values are given as percentage of cell viability. *p < 0.05, significance of difference compared with control. Fot. 1. BT cells (control cells), magnification of ×100, Fot. 2. Cythopathic effect of BVDV-1 in BT cells, magnification of ×100, Fot. 3. HEp-2 cells (control cells), magnification of ×100, Fot. 4. Cythopathic effect of HSV-1 in HEp-2 cells, magnification of ×200, Fot. 5. Cythopathic effect of HSV-1 in HEp-2 cells, magnification of ×630—new viruses released from cells

In order to determine the efficacy of chromium(III) and iron(III) against HSV1 in vitro, we first analyzed its cytotoxicity on cultured HEp-2 cells. While, to determine the efficacy of chromium(III) and iron(III) against BVDV in vitro, we first analyzed its cytotoxicity on cultured BT cells. Cytotixicity assays are fundamental for the initial phases of antiviral substances development because they define the concentrations to be used. The assesment of the cytotoxicity of new substances is usually performed by cell viability. The MTT assay is probably one of the most widely used assay (Abdel-Rahman et al. 2016). In our study the cytotoxicity of the tested microelements was evaluated by this assay and the IC_50_ values are expressed in μM. The cytotoxicity results of chromium(III) and iron(III) are presented in Figs. 1 and 2. The MTT assay demonstrated a low toxicity of microelements used on HEp-2 cells and BT cells. The IC_50_ for chromium chloride was 1100 μM for Hep-2 cells and 1400 μM for BT cells. The IC_50_ for iron chloride was 1200 μM for Hep-2 cells and more than 1400 μM for BT cells. These results are presented in Table 1.

**Table 1:** Cytotoxicity of chromium chloride and iron chloride. Inhibitory concentration (IC_50_, μM)

	HEp-2 line	BT line
CrCl_3_ × 6H_2_O	1100	1400
FeCl_3_ × 6H_2_O	1200	>1400

### Antiviral activity of chromium chloride and iron chloride

All samples were observed under microscope. When cythopathic effect was observed, RNA was extracted from supernatant of infected BT cells (Fot. 1 and 2) and DNA was extracted from supernatant of infected HEp-2 cells (Fot. 3, 4, 5).

**Fig. 3:**
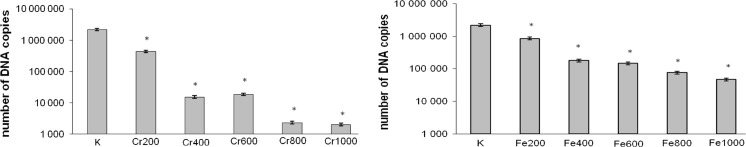
Effect of chromium chloride or iron chloride on HSV-1 replication. *p < 0.05, significance of difference compared with control

**Fig. 4:**
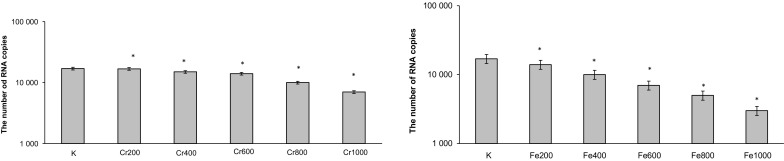
Effect of chromium chloride or iron chloride on BVDV replication. *p < 0.05, significance of difference compared with control

The data of the antiviral activity of chromium chloride and iron chloride used separately are demonstrated in Figs. 3 and 4. Chromium chloride and iron chloride show antiviral effect in all used concentrations which did not affect the cell viability. Figure 3 shows the in vitro effects of iron(III) and chromium(III) on number of copies of DNA. Moreover, iron(III) and chromium(III) used separately decrease the number of copies of RNA in all concentrations (Fig. 4).

**Fig. 5:**
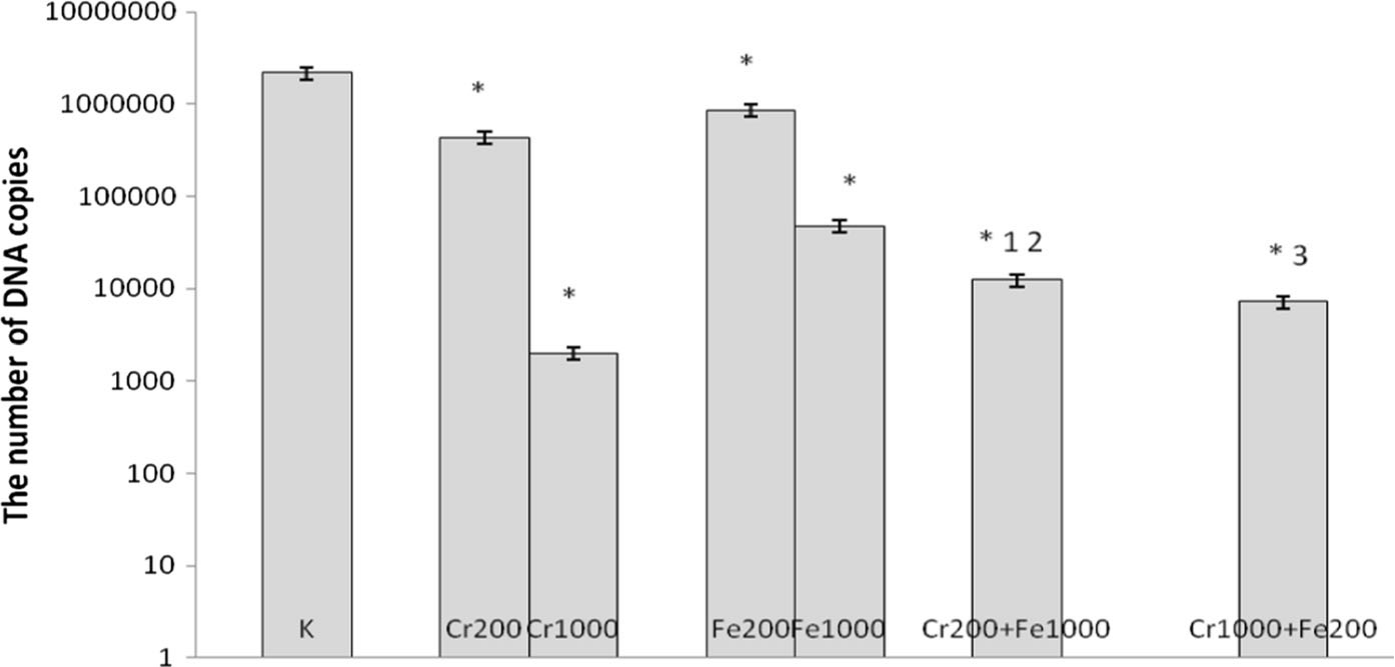
Effects of chromium chloride and iron chloride on number of copies of DNA. *p < 0.05, significance of difference compared with control. *1* p < 0.05, significance of difference compared with chromium chloride at concentration of 200 μM; *2* significance of difference compared with iron chloride at concentration of 1000 μM; *3* p < 0.05, significance of difference compared with iron chloride at concentration of 200 μM

**Fig. 6:**
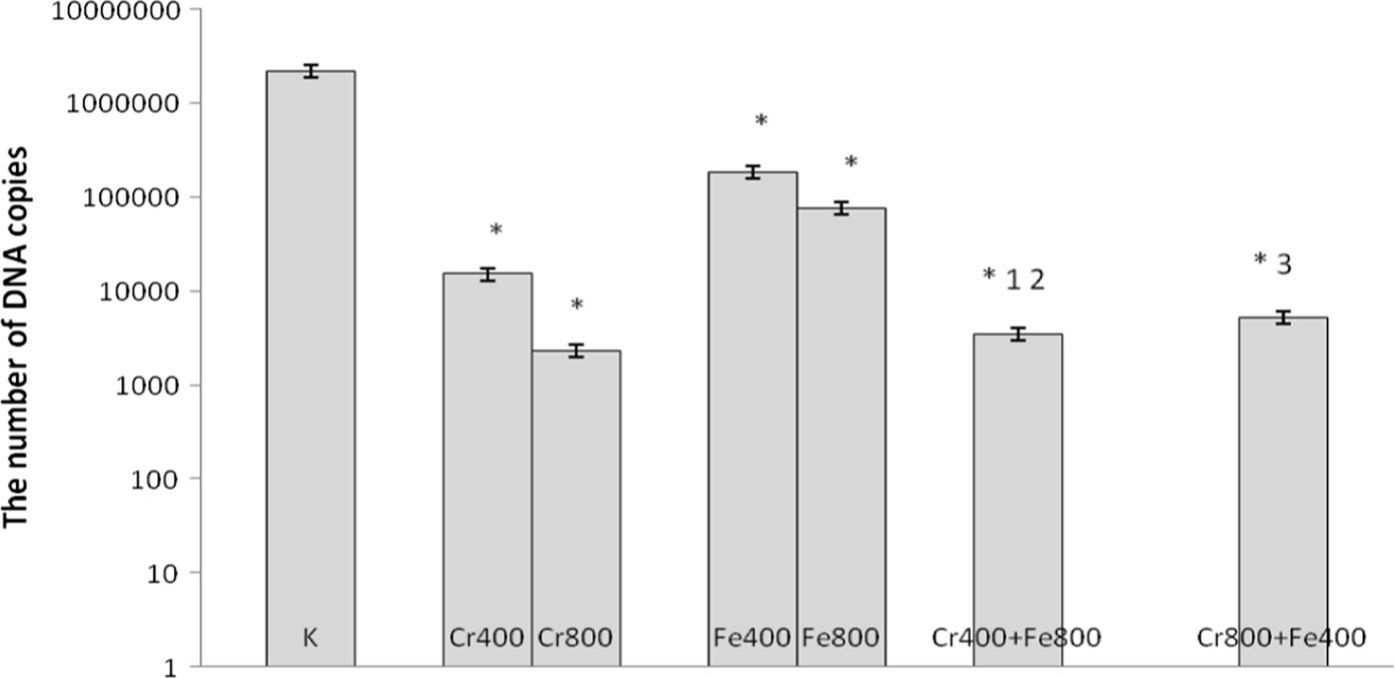
Effects of chromium chloride and iron chloride on number of copies of DNA. *p < 0.05, significance of difference compared with control. *1* p < 0.05, significance of difference compared with chromium chloride at concentration of 400 μM; *2* p < 0.05, significance of difference compared with iron chloride at concentration of 800 μM; *3* p < 0.05, significance of difference compared with iron chloride at concentration of 400 μM

In cultures simultaneously treated with 200 μM of CrCl_3_ and 1000 μM of FeCl_3_, 1000 μM of CrCl_3_ and 200 μM of FeCl_3_, 400 μM of CrCl_3_ and 800 μM of FeCl_3_, 800 μM of CrCl_3_ and 400 μM of FeCl_3_ a decrease in number of copies of DNA was observed compared with control cells and cells incubated with chromium(III) at concentrations 200 and 400 μM and iron(III) used separately (Figs. 5, 6).

**Fig. 7:**
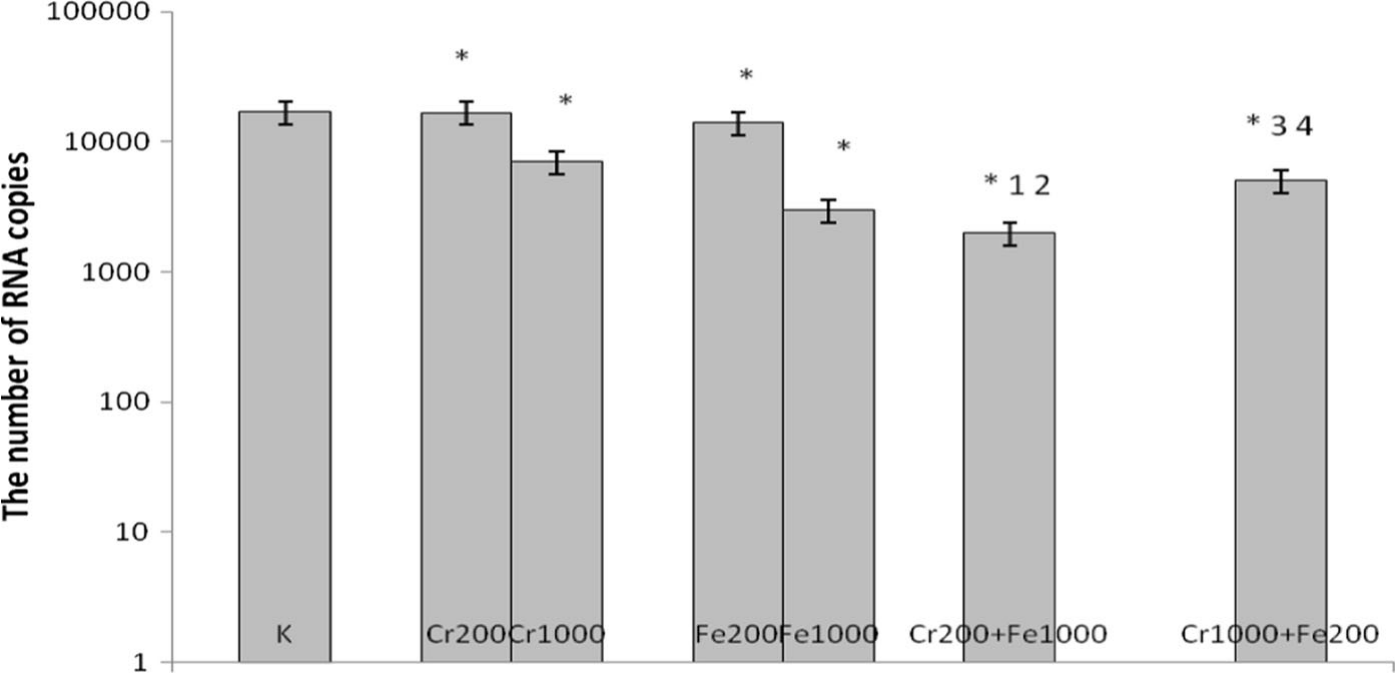
Effects of chromium chloride and iron chloride on number of copies of RNA. *p < 0.05, significance of difference compared with control. *1* p < 0.05, significance of difference compared with chromium chloride at concentration of 200 μM; *2* p < 0.05, significance of difference compared with iron chloride at concentration of 1000 μM; *3* p < 0.05, significance of difference compared with chromium chloride at concentration of 1000 μM; *4* p < 0.05, significance of difference compared with iron chloride at concentration of 200 μM

**Fig. 8:**
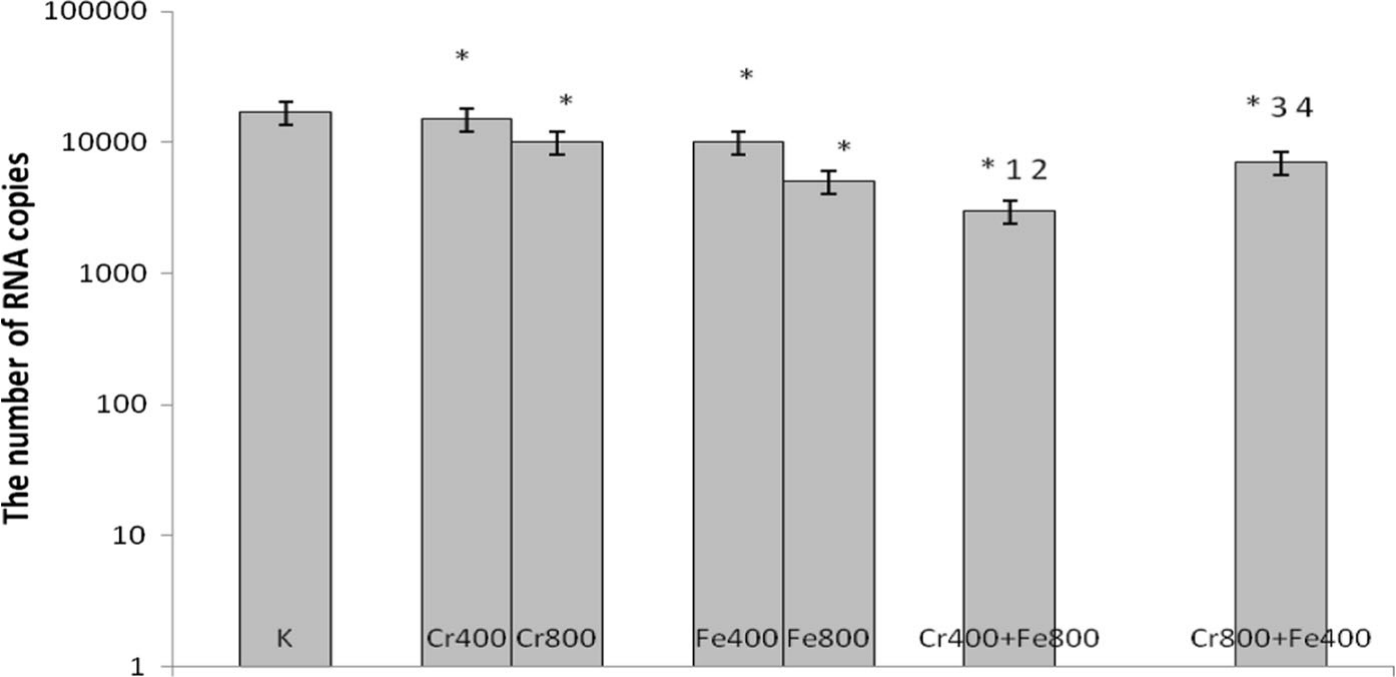
Effects of chromium chloride and iron chloride on number of copies of RNA. *p < 0.05, significance of difference compared with control. *1* p < 0.05, significance of difference compared with chromium chloride at concentration of 400 μM; *2* p < 0.05, significance of difference compared with iron chloride at concentration of 800 μM. *3* p < 0.05, significance of difference compared with chromium chloride at concentration of 800 μM; *4* p < 0.05, significance of difference compared with iron chloride at concentration of 400 μM

In cultures simultaneously treated with 200 μM CrCl_3_ and 1000 μM FeCl_3_, 1000 μM CrCl_3_ and 200 μM FeCl_3_, 400 μM CrCl_3_ and 800 μM FeCl_3_, 800 μM CrCl_3_ and 400 μM FeCl_3_ a decrease in number of copies of RNA was observed compared with control cells and cells incubated with chromium(III) and iron(III) used separately (Figs. 7, 8).

## Disscusion

The relationship between microelements in in vitro and in vivo experiments has attracted attention of many investigators. Nutrient-nutrient interactions may negatively or positively affect the cell viability and replication of bacteria or viruses.

The concentrations of chromium chloride and iron chloride for these studies were chosen on the basis of other reports (Mazzotti et al. 2001, 2002) and our earlier investigations. Our previous experiments have shown that both of them slightly stimulated cell proliferation (Terpiłowska and Siwicki 2009, 2010). Our previous investigations have shown that chromium(III) and iron(III) statistically increase IL-1α at the concentrations of 50 and 500 μM, and that they decrease IL-6 concentration when compared with control cells. Simultaneous treatment with chromium and iron suggests the synergistic interaction between these elements (Terpiłowska and Siwicki 2012).

At the molecular level the most promising mechanisms of antiviral intervention may involve: prevention of the formation of the virus protein’s active configuration (e.g. by blocking normal homodimer or heterodimer formation) or elimination of enzymatic function by blockage or distortion of the active site through strong or irreversible binding of a specific antimetabolite; or subversion of the protein’s normal function towards lethal synthesis—for example by provision of an innocuous substrate for conversion in the virus infected cells into a DNA chain terminator or destabilizer or antimetaboite (Subak-Sharpe and Darga 1998).

The first antivirals, 5-Iodo-2′-deoxyiuridine (IDU) and 5-Trifluoromethyl-2′-deoxyuridine (TFT), date from the 1960s, and until today, they have been still used. The first antiviral compound used in the systemic treatment of herpesvirus infections was vidarabine. It was then superseded by acyclovir, which appeared to be more selective in its activity against HSV because of a specific phosphorylation by the virus-induced tymidine kinase (TK) (De Clerq 2013). However, a serious problem in the use of acyclovir is drug resistance in patients. Moreover, these drugs are expensive and several patients with frequent attacks may not be able to afford the cost of long-term treatment (Khan et al. 2005). There is a great interesting possibility to develop efficacious antiviral compounds being natural products with low toxicity that are well tolerated (Glatthaar-Salmüller et al. 2011). As an alternative, naturally occurring substances found in plants and fruit could be cheaper and more efficacious to use (Danaher et al. 2011). These natural substances, which are food component, are easily consumed to prevent viral infections. The extracts from seeds, plants, roots and fruit have been shown to have antiviral activities (Danaher et al. 2011).

There are several in vitro investigations to study the anti-herpetic activities of plant/herbal extracts or plant-derived molecules. The anti-HSV activity of moronic and betulonic acids from the herbal extract of *Rhus javanica* has been reported. Moreover, the aqueous extracts and pure compounds of *Plantago major* L have been reported as antiviral (on HSV-1, -2 and adenoviruses-3, -8, 11) agent (Khan et al. 2005). Polyphenols derived from plants have been shown to have antiviral activity. Especially, the flavonoids, galangin, quercitin, procyanidin, and pelargonidin are found to be virucidal against HSV (Danaher et al. 2011). Danaher et al. (2011) shows that blackberry extract has strong antiviral- anti HSV effects that interfere with absorption or entry into host cells and some intracellular activity. Glatthaar-Salmüller et al. (2011) reported concentration-dependent antiviral activity (EC_50_ between 13.8 and 124.8 μg/ml) of Sinupret against RNA and DNA viruses. Sinupret is herbal medicine product made from *Gentian root*, *Primula flower*, Sorrel herb and Verbena herb. Moreover, metal nanoparticles were found to be virucidal against DNA and RNA viruses. Metal nanoparticles such as silver and gold have demonstrated efficient inhibitory activity against Human Immunodeficiency Virus, Hepatitis B Virus, influenza virus and Peste des petits ruminants virus (Hang et al. 2015). Hang et al. (2015) demonstrated that cuprous nanoparticle (at cytotoxic concentration) significantly inhibited Hepatitis C virus infection in the HCVcc/Huh7.5.1 cell culture system. Furthermore, it was indicated that cuprous oxide, sulphide, iodide and chloride have highly efficient antiviral activities- on bacteriophage QB (Sunada et al. 2012). Nickel was another tested element. The antiviral activity of Ni-chitosan microcomposite affects the free virus adsorption to the cells by binding to the VP1 protein of enterovirus 71 (Lin and Chang 2014). Investigation conducted by Abdel-Rahman et al. (2016) shows that nanoCr(III) and nanoFe(II) complexes inhibit HSV and Tobacco Mosaic Viruses (TMV) replication. Minimum inhibitory concentration required to reduce virus-induced cytopathogenicity by 50% was 126.4 μg/ml for nanoCr(III) and 12.3 μg/ml for nanoFe(II) against HSV. Minimum inhibitory concentration required to reduce virus-induced cytopathogenicity by 50% was 70 μg/ml for nanoCr(III) and 0,83 μg/ml for nanoFe(II) against TMV.

Possible mechanisms of the antiviral activity include: interaction with the viral surface, interference with the viral attachment, inhibition of virus penetration into the cell, interaction with the viral genome, inhibition of genome replication, inhibition of protein synthesis and inhibition of assembly and release of virions (Hang et al. 2015). In this study, we demonstrated a decrease in number of copies of DNA of HSV after a 48 h of incubation or RNA of BVDV after 5 days of incubations. Here we report the antiviral activities of chromium(III) and iron(III) against HSV-1 and BVBV. For HSV-1, synergistic effect was found at higher concentrations of chromium chloride, i.e. 800 and 1000 μM combined with iron chloride (200, 400, 800 and 1000 μM). For BVDV1, synergistic effect was found for chromium chloride combined with iron chloride. The mechanism of antiviral synergism between chromium and iron has not been determined. It has been demonstrated that Cr(III) compounds can bind directly to DNA or RNA in vitro, forming Cr-DNA adducts and DNA–DNA crosslinks (O’Brien et al. 2003). Moreover, Cr(III) has been shown to be able to increase the catalytic activity and decrease the fidelity of DNA polymerase (Galaris and Evangelou 2002). Moreover, excess free iron(III) promotes the formation of reactive oxygen species (ROS), which attack cellular nucleic acids (Andrews 2000, 2005). It is assumed that chromium(III) and iron(III) used separately and simultaneously may interact with viral DNA or RNA and this interaction may result in interaction with the viral genome and blocking the genome replication.

Chromium(III) and iron(III) are essential trace elements in the human body, which play a crucial role in the biochemistry of all living organisms (Hang et al. 2015). Nutrition plays an important role in the development and also in the prevention of cancer, cardiovascular diseases, and diabetes. Chromium(III) and iron(III) are trace elements necessary for growth and normal functioning of cells. We observed significant inhibitory effects of chromium(III) and iron(III) against HSV and BVDV, at non cytotoxic concentrations in the HEp-2 and BT cells. Thus, these problems still demand a lot of investigation. Future research is warranted to assess their potential for practical use in clinical settings.
